# Two Cases of Progressive Familial Intrahepatic Cholestasis Type 2 Presenting with Severe Coagulopathy without Jaundice

**DOI:** 10.1155/2014/185923

**Published:** 2014-06-02

**Authors:** Eric Tibesar, Christine Karwowski, Paula Hertel, Ann Scheimann, Wikrom Karnsakul

**Affiliations:** ^1^Division of Pediatric Gastroenterology, Johns Hopkins University School of Medicine, 600 N. Wolfe Street, Baltimore, MD 21287, USA; ^2^Division of Pediatric Gastroenterology, Texas Children's Hospital, 6621 Fannin Street, Houston, TX 77030, USA

## Abstract

Progressive familial intrahepatic cholestasis (PFIC) type 2 results from a mutation in the bile salt exporter pump, impeding bile acid transport. Patients usually present with jaundice, pruritus, growth failure, and fat soluble vitamin deficiencies. We present two patients diagnosed with PFIC type 2 due to severe coagulopathy and bleeding without jaundice.

Case  A is a healthy 5-month-old female who presented to a local hospital with scratching to the point of bleeding and ecchymoses on her abdomen, back, and legs. She had no history of jaundice since birth and jaundice was not noted at several doctors' visits prior to this presentation. Physical examination showed neither icterus nor hepatosplenomegaly. Initial labs demonstrated AST 223 U/L, ALT 334 U/L, total bilirubin 3.4 mg/dL, direct bilirubin 2.8 mg/dL, GGT 33 U/L, partial thromboplastin time (PTT) 90.8 seconds, prothrombin time (PT) > 120.0 seconds, and INR > 13.7. She received a one-time intravenous vitamin K with repeat INR of 1.0 and then was admitted for further evaluation. Of note, she was treated with IV cefotaxime at the outside hospital due to a positive urine culture for* Escherichia coli*, and this was continued for a full seven-day course.

Cholestasis persisted with a peak direct bilirubin of 7.5 mg/dL. An abdominal ultrasound showed normal hepatic echotexture and biliary system without focal lesions. Infectious work-up included negative serologies for CMV, EBV, HIV, HSV, HCV, and HBV. As vitamin K deficiency was thought to be from cholestasis related malabsorption, fat soluble vitamin studies were performed and revealed a vitamin D (25-OH) level of <5 ng/mL, normal vitamin A, and an alpha-tocopherol level of 0.8 mg/L. Total serum bile acids were elevated at 205.3 umol/L (normal; 4.5–19.2 umol/L). Percutaneous liver biopsy revealed mild chronic portal inflammation, periportal fibrosis, ballooning hepatocytes, significant cholestasis, and early bile duct loss with ductular proliferation (see Figures 1 and 2). No bile salt exporter pump (BSEP) staining was performed. Serum sent for genetic evaluation revealed heterozygous mutations in the ABCB11 gene (c.908(+1)G>A/c.3692 G>A (R1231Q)), confirming the diagnosis of progressive familial intrahepatic cholestasis (PFIC) type 2.

Following discharge, she suffered from intractable pruritus despite the use of ursodiol, rifampin, cholestyramine, and hydroxyzine. She underwent an internal ileal diversion at 12 months with no relief of significant pruritus and continued presence of high total bile acid level of 147.4 umol/L and subsequently an internal biliary diversion at 15 months, again without relief of pruritus and presence of even higher total bile acid level of 239.2 umol/L. Growth had been normal, but the patient had low vitamin E and D levels, despite large dose supplementation. She was listed for living related donor liver transplant at this point.

Case B is a 14-year-old male, clinically diagnosed with Alagille syndrome as an infant due to cholestasis without evidence of jaundice and a liver biopsy that showed paucity of intrahepatic bile ducts. Past medical history is significant for intracerebral hemorrhage during infancy, presumably due to vitamin K deficiency, leading to life-long seizure disorder and residual left-sided hemiparesis. He was treated with phenytoin for seizure control and followed up closely by the pediatric liver service due to concerns about fat soluble vitamin deficiencies, cholestasis, and poor growth.

At the age of 14, he presented to an outside emergency room with leg pain and increased bruising. Jaundice was not noted. Labs showed PT of 40 seconds and PTT of 120 seconds. He received a one-time intramuscular dose of 30 mg vitamin K, and repeat labs showed PT of 16.7 seconds and PTT of 35.0 seconds. Total bilirubin was 0.6 mg/dL and GGT was normal (see Table 1 for comparison to Case A). No antibiotics were given and he was discharged home the next day, with close follow-up arranged in the pediatric GI clinic.

Over the following 15–18 months, he developed jaundice with worsening cholestasis and increased pruritus. At the age of 16, due to increased jaundice, he underwent endoscopic retrograde cholangiopancreatography (ERCP) and percutaneous liver biopsy. The ERCP was normal. Liver biopsy showed marked canalicular and hepatocellular cholestasis, with mild to moderate portal and lobular fibrosis including BSEP staining (see Figures 3 and 4). There was no paucity of intrahepatic bile ducts, with findings inconsistent with Alagille syndrome. Because of continued jaundice and cholestasis, genetic testing was done, showing mutations in the ABCB11 gene (c.890A>G; p.Q297G/c.2343+1 G>T), confirming the diagnosis of PFIC type 2 [1]. Case B reached adult age without transplantation.

*Discussion*. These two cases demonstrate an unusual presentation of PFIC type 2 in that both had severe coagulopathy without the presence or the history of jaundice in their clinical manifestations. Although both patients eventually developed a more obvious feature of jaundice, their initial presentations of cholestasis and coagulopathy in the absence of jaundice were concerning enough to warrant further investigations in order to provide proper management. In a recent analysis of presenting signs and symptoms of patients with BSEP mutations, the most common finding was jaundice (73% of patients), with 9% of patients presenting with manifestations of vitamin deficiency [2]. Of those 9% of patients, 58% presented without clinical signs of jaundice, although the exact vitamin deficiency and presence of clinical bleeding were not mentioned [2]. There was no mention of any patient specifically presenting with coagulopathy or clinical bleeding, corrected with vitamin K, in the absence of jaundice.

Patients with PFIC type 2 can be diagnosed based on mutations in the ABCB11 gene, which encodes the BSEP protein. To date, there are more than 100 mutations that have been discovered in the ABCB11 gene, leading to a 70% reduction or complete absence of bile salts removed from the liver [3]. Two of the more common mutations that have been found include the missense mutations E297G and D482G, found in up to 30% of European patients with PFIC type 2 [4]. A retrospective analysis of 84 patients with ABCB11 gene mutations found that 61% of them had one or two alleles with these more common missense mutations [2]. Ten were noted to have coagulopathy from vitamin deficiency upon presentation, with three being noted to have the D482G mutation [2]. Both patients in this report had genetic analysis, without evidence of either of the two more common mutations.

In Case A the first mutation is c.3692 G>A in exon 27 [5]. The predicted protein effect is R1231Q, which does not lead to an abnormal splice site but causes a missense mutation. This results in an immature protein or delayed maturation of BSEP, presumably lowering its function [6]. This mutation was also reported in 2 patients who had homozygous and compound heterozygous mutations [5, 7]. The patient with homozygous mutations developed severe fibrosis and underwent liver transplantation at the age of 2.9 years. The other mutation at the splice site, c.908+1G>A in intron 9 of* ABCB11*, has also been reported by Strautnieks et al. [5]. In that study, this mutation was found in a compound heterozygote for c.908+1G>A with another common missense mutation c.1445A>G. The c.1445A>G is predicted to cause p.Asp482Gly; however, its true effect is aberrant splicing which leads to translation of a truncated protein [6]. The progression of liver disease in this patient was actually slow.

In Case B, his first mutation is 890 A>G in exon 9 [5]. The predicted protein effect is E297G or p.Glu297Gly. The other mutation is c.2343+1 G>T which is a mutation at splice site, 5′ intron 19, a novel splice site change. This compound heterozygous mutation was reported in Case B's sister who developed persistent cholestasis and bridging fibrosis at the age of 2 years, biliary cirrhosis at the age of 3 years, and cholangiocarcinoma and died at the age of 4 [1].

There are technical challenges to the exploration of the mRNA consequences of the splice site mutations by sequencing the corresponding cDNA. Since expression of BSEP protein in extrahepatic tissues is low, the only way to get sufficient mRNA is sampling the explanted liver and conserving the RNAse inhibitors immediately at the time of surgery, which was not feasible in both cases. We can assume that Case B must have similar mutations as his sister whose mRNA sequencing was performed and published by Scheimann et al. [1]. On the other hand, from a clinical perspective, one would assume that homozygotes of c.3692 G>A would manifest a more severe presentation compared to a compound heterozygote of c.908(+1)G>A/c.3692 G>A, similar to Case A. In theory, the expected result of c.908+1G>A could be exon 9 skipping with deletion, frameshift mutation resulting in a premature stop codon and protein truncation. However, less likely events include skipping of more than one exon or cryptic splice site activation. More importantly, the absence of the wild type transcript, which originates from the allele carrying the splice site mutation, is rather complicated in a compound heterozygote, especially with a normally transcribed allele carrying a 2.3 kb downstream missense mutation. In one report, biallelic BSEP mutations were described in PFIC type 2 children with a tendency to have lower bile acid concentration and lower age at liver transplantation than those with one truncating and one missense mutation; an expected correlation with a severe mutation with vitamin malabsorption and deficiencies was not reported [8]. Perhaps there is yet more to learn about genotype and phenotype association to understand the molecular function of the BSEP protein.

In this report we conclude that patients who present with cholestasis and significant coagulopathy that respond to vitamin K should have congenital liver diseases such as PFIC included in their differential diagnosis though cholestasis may not be pronounced as a common clue in the diagnosis. Genetic analysis for mutations in the ABCB11 gene can aid in diagnosis after ruling out common causes of cholestasis. Further study in larger cohorts of PFIC type 2 populations may reveal more specific genotype-phenotype correlations.

## Figures and Tables

**Figure 1:**
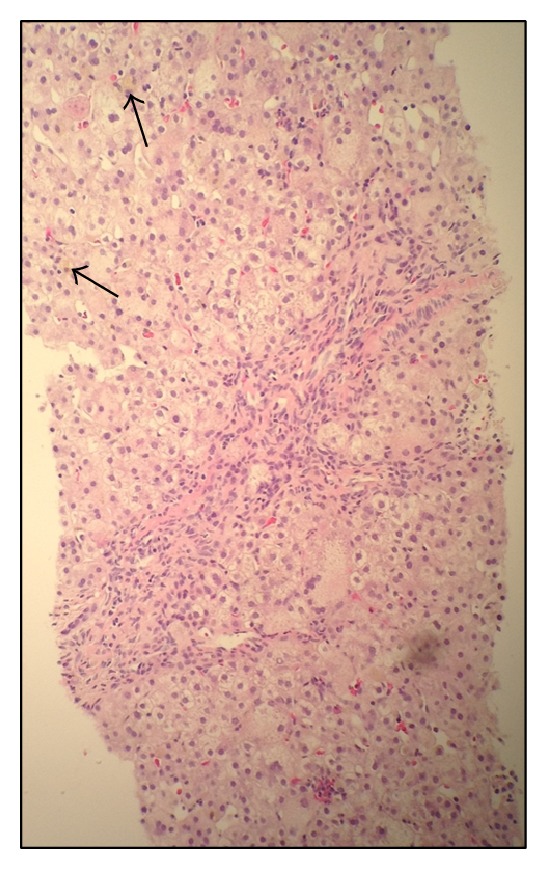
Liver biopsy at 100x magnification showing bile duct plugs (arrows) along with multinuclear giant/hydropic cells.

**Figure 2:**
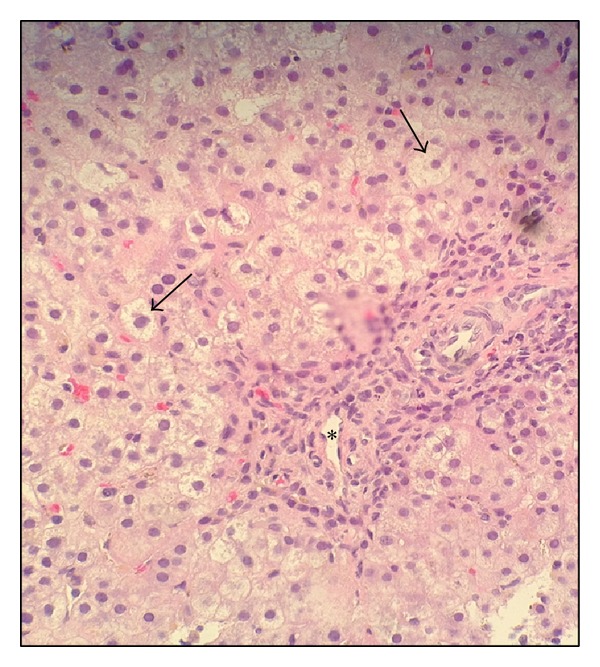
Liver biopsy at 200x magnification showing ballooning hepatocytes (arrows) with inflammation around the portal vein (*).

**Figure 3:**
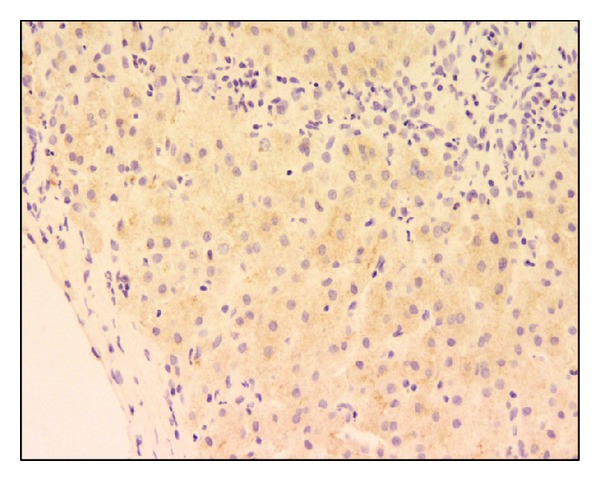
Liver biopsy of Case B, at 20x magnification showing staining for BSEP protein.

**Figure 4:**
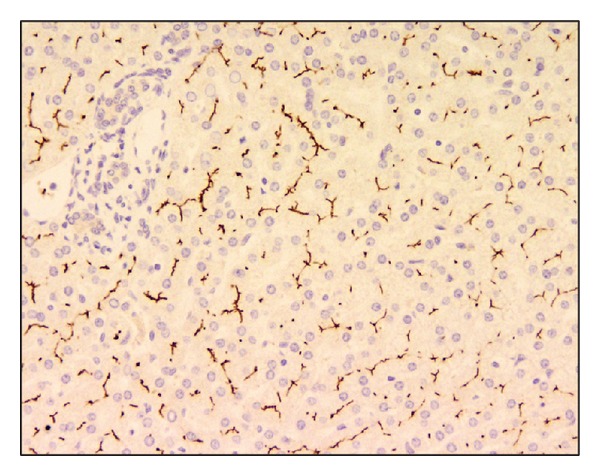
Liver biopsy at 20x magnification, used as a positive control for biopsy in Figure 3. BSEP protein stains are brown.

**Table 1:** Initial presenting clinical and laboratory data.

Case	Age at diagnosis	ALT (U/L)	Total bilirubin (mg/dL)	Direct bilirubin (mg/dL)	Serum bile acids (umol/L)	GGT (U/L)	Prothrombin time (seconds)	INR	Vitamin D (25-OH) (ng/mL)
A	5 months	334	3.4	2.8	205.3	33	>120.0	>13.7	<5
B	14 years	57	0.6	0.1	n/a	35	40	4.2	n/a

Values marked n/a mean that no data was available for that patient. Normal reference values: ALT, 0–31 U/L; total bilirubin, 0.1–1.2 mg/dL; direct bilirubin, 0.0–0.4 mg/dL; serum bile acids, 4.5–19.2 umol/L; GGT, 8–51 U/L; prothrombin time, 9.4–11.6 seconds; INR, 0.9–1.1; vitamin D (25-OH), 32–100 ng/mL.

## Abbreviations

PTT: Partial thromboplastin time
PT: Prothrombin time
BSEP: Bile salt exporter pump
PFIC: Progressive familial intrahepatic cholestasis.
